# MicroRNA-Sequence Profiling Reveals Novel Osmoregulatory MicroRNA Expression Patterns in Catadromous Eel *Anguilla marmorata*

**DOI:** 10.1371/journal.pone.0136383

**Published:** 2015-08-24

**Authors:** Xiaolu Wang, Danqing Yin, Peng Li, Shaowu Yin, Li Wang, Yihe Jia, Xinhua Shu

**Affiliations:** 1 Jiangsu Key Laboratory for Biodiversity and Biotechnology, College of Life Sciences, Nanjing Normal University, Nanjing, China; 2 Faculty of Medicine, Dentistry and Health Sciences, The University of Melbourne, Parkville VIC 3010, Australia; 3 Department of Life Sciences, Glasgow Caledonian University, Cowcaddens Road, Glasgow, United Kingdom; 4 Co-Innovation Center for Marine Bio-Industry Technology of Jiangsu Province, Lian Yungang, China; Sabanci University, TURKEY

## Abstract

MicroRNAs (miRNAs) are a class of endogenous small non-coding RNAs that regulate gene expression by post-transcriptional repression of mRNAs. Recently, several miRNAs have been confirmed to execute directly or indirectly osmoregulatory functions in fish via translational control. In order to clarify whether miRNAs play relevant roles in the osmoregulation of *Anguilla marmorata*, three sRNA libraries of *A*. *marmorata* during adjusting to three various salinities were sequenced by Illumina sRNA deep sequencing methods. Totally 11,339,168, 11,958,406 and 12,568,964 clear reads were obtained from 3 different libraries, respectively. Meanwhile, 34 conserved miRNAs and 613 novel miRNAs were identified using the sequence data. MiR-10b-5p, miR-181a, miR-26a-5p, miR-30d and miR-99a-5p were dominantly expressed in eels at three salinities. Totally 29 mature miRNAs were significantly up-regulated, while 72 mature miRNAs were significantly down-regulated in brackish water (10‰ salinity) compared with fresh water (0‰ salinity); 24 mature miRNAs were significantly up-regulated, while 54 mature miRNAs were significantly down-regulated in sea water (25‰ salinity) compared with fresh water. Similarly, 24 mature miRNAs were significantly up-regulated, while 45 mature miRNAs were significantly down-regulated in sea water compared with brackish water. The expression patterns of 12 dominantly expressed miRNAs were analyzed at different time points when the eels transferred from fresh water to brackish water or to sea water. These miRNAs showed differential expression patterns in eels at distinct salinities. Interestingly, miR-122, miR-140-3p and miR-10b-5p demonstrated osmoregulatory effects in certain salinities. In addition, the identification and characterization of differentially expressed miRNAs at different salinities can clarify the osmoregulatory roles of miRNAs, which will shed lights for future studies on osmoregulation in fish.

## Introduction

MicroRNAs (miRNAs), a class of small non-coding RNAs with the length of 18–26 nt, can post-transcriptionally regulate the expression of endogenous genes [1,2]. Due to the imperfect base pairing with 3’-untranslated region (3’-UTR) of target mRNAs, miRNAs can mediate translational repression or mRNA degradation [3]. Since the identification of the first miRNA *lin-4* in developmental stages of *Caenorhabditis elegans*, numerous miRNAs have been subsequently identified in animals and plants [4]. Many miRNAs are evolutionarily conserved with the “seed” sequence, and some miRNAs exhibit tissue-and/or time-specific expression [2]. One miRNA may regulate hundreds of target mRNAs, whereas one gene may contain multiple binding sites of miRNAs, thus resulting in a potential and complex regulatory network [5–8]. Functional studies have indicated that miRNAs can participate in the regulation of different cellular processes [5,9].

Maintaining cell volume and structural dynamics is vital for organisms during cellular life [10], and is especially crucial for teleost, because maintaining water and ion homeostasis in their gills is indispensable to osmotic adjustment during migration. Hundreds of cellular events can be observed during osmotic stress in teleost such as alteration in the activities of cellular receptors and reorganization of the cellular cytoskeleton architecture [10,11]. The major regulators of osmotic stress appear to be involved in the change of external ion contents or internal hormonal levels in fish, but it is still unknown which factors or molecules are predominantly influential to osmoregulatory mechanisms. Several studies have been conducted to explore the potential factors for osmoregulation. Osmotic stress transcription factor 1 (OSTF1) is an important molecule for osmoregulation as a putative transcriptional regulator in early hyperosmotic regulation [12]. OSTF1 was first identified in *Oreochromis mossambicus* [13]. Subsequently, the OSTF1 of Japanese eel *Anguilla japonica* has been successfully cloned and shared 84% DNA homology with the OSTF1 of tilapia [14]. The number of ion channels or transporters can be regulated by increasing or decreasing the transcription and/or translation of corresponding genes [15], such as Na^+^/K^+^/2Cl^-^ cotransporter (NKCC) and cystic fibrosis trans-membrane conductance regulator (CFTR). Cl^-^ channels can be up-regulated in fish gill after sea water acclimation [16]. Recently it has been reported that signalling pathways play an important role in osmotic stress, such as myosin light chain kinase (MLCK), focal adhesion kinase (FAK), and mitogen activated protein kinase (MAPK) pathways [17–21]. It is also well known that the functional evidences of glucocorticoid receptors and calcium sensing receptors are illustrated in zebrafish by morpholino knockdown technology [22,23]. Moreover, hormones including growth hormone (GH), insulin-like growth factor-1 (IGF-1), thyroid-stimulating hormone (TSH) and prolactin (PRL) play important roles in the osmoregulation of fish species [24,25]. Although several molecules, pathways and hormones related to osmoregulation have been reported previously, the miRNAs involved in osmoregulation are still less reported. For instance, it is highlighted that miR-200a and miR-200b from miR-8 family in zebrafish embryos reveal an obvious impact on Na^+^/H^+^ exchanger; concurrently, an increase in the osmotic pressure sensitivity can result in Na^+^ accumulation in ionocytes [26]. In addition, *in vivo* trials have demonstrated that down-regulation of miR-429 in tilapia could result in an substantial increase in OSTF1 expression, which is responsible for osmosensory signal transduction [27]. The loss of miR-30c function can lead to an inability to respond to osmotic stress that directly regulates *hsp70* expression by targeting *hsp70* 3’-UTR [28]. IGF-1 is also identified as the target gene of miR-206 in tilapia and IGF-1 treatment can up-regulate the expression of transporters such as Na^+^, K^+^-ATPase, and NKCC [29,30]. Through those studies, some effects of miRNAs on osmoregulation have been clarified, but a complicated molecular regulatory network remains unclear.

*Anguilla marmorata*, one of the quint essential catadromous fish, also known as marbled eel, is a tropical eel widely spread across tropical and subtropical oceans and associated with fresh water systems. *A*. *marmorata* is also placed in the International Union for Conservation of Nature (IUCN) Red List of threatened species, and is regarded as species under second-class protection in China, due to the excessive fishing under the stimulation of its high commercial value, especially in Asian and Southeast Asian fish markets [31]. During the continental growth stages, the eels have frequently encountered the osmoadaptation challenge during migrating reciprocally between fresh water and sea water [32]. The juvenile eels are usually born in the sea, and then migrate to fresh water for primary growth, following by the return to the sea for the reproduction during the adult period [33]. Thus, the transition along gradient salinity throughout life requires the eels to have a well-established osmoregulatory system. Even though the molecular mechanisms of osmoregulation have been addressed from different aspects in other close species of the eels, the information on how miRNAs complete osmostress-induced responses through the alternation of osmospecific gene expression in osmoregulatory organs such as gills in the marbled eels are still limited. We hypothesize that miRNAs contribute to differential expression pattern in the body of marbled eels in various salinities. We aim to identify differentially expressed miRNAs in different salinities, and most importantly, to reveal the role of miRNAs in osmoregulation in marbled eels. Our data will provide referential information for future studies on the aquaculture and conservation of marbled eels.

## Materials and Methods

### Ethics statement

The experiments were conducted on *A*. *marmorata* that is regarded as species under second-class state protection in China. All experiments were performed according to the Guideline for the Care and Use of Laboratory Animals in China. This study was also approved by the Ethics Committee of Experimental Animals at Nanjing Normal University. The location is not privately-owned or protected in anyway. All eels were provided by Hainan Wenchang Jinshan eel technology limited company which has obtained The People's Republic of China aquatic wild animal catching permit from Ministry of Agriculture of The People's Republic of China since 2004 (Approval number: National Fishery Resources and Environmental Protection 2004; 13).

### Collection of *A*. *marmorata* samples

For Illumina sequencing, 52 juvenile individuals of *A*. *marmorata* were captured from Wanquan River in Hainan Island, China (19°08’17N, 110°15’46E). After acclimatized in our laboratory for 1 week, 18 of 52 eels with similar size and weight were exposed to different salinities for 15 days, including 6 individuals in fresh water (FW, 0‰ salinity), 6 in brackish water (BW, 10‰ salinity) and 6 in sea water (SW, 25‰ salinity). Each individual was dissected on ice and its gill tissues were immediately frozen in liquid nitrogen and stored at -80°C until RNA isolation. Totally 18 gill tissues were assigned to 3 groups, each has two biological replicates (assigned as P1 and P2), and each replicate consisted of three different individual gill tissues.

For miRNA time-course expression experiment, twenty-seven juvenile individuals of *A*. *marmorata* were provided by the same company as described above. The experimental eels were primarily placed in FW (0 h, salinity of 0‰) and the gills tissues were isolated (n = 3), and then the salinity was gradually increased by 3‰ everyday until it reached up to 10‰ (BW) or 25‰ (SW). In order to determine the temporal expression of miRNAs in salinity adaptation groups, gill tissues were collected from three eels in each treated group at 1, 6, 12 and 24 h after the desired salinity was established (n = 3). During sampling process above, experimental eels were anaesthetized with a solution of 0.05% 2-phenoxyethanol (Sigma-Aldrich, St Louis, MO, USA).

Total RNA of the gill tissues mentioned above were extracted by High Purity RNA Fast Extract Reagent (Bioteke, Beijing, China) according to the manufacturer’s protocol. The same reagent was using in subsequent experimental sampling. The quantity of total RNA was measured by using NanoDrop 2000 (Thermo Fisher Scientific, Waltham, MA, USA), and its integrity was examined in 1.0% agarose gel.

### sRNA library construction and sRNA deep sequencing

After sRNAs with 15–33 nt in length were isolated from 1 μg total RNA by size fractionation in a 15% TBE urea polyacrylamide gel, the purified sRNAs were then ligated to 3′ adaptors and 5′ adaptors (Illumina, San Diego, CA, USA). Briefly, the first strand of cDNA was synthesized with reverse transcription. Subsequently, the synthesized cDNAs were subjected to 15 PCR cycles using primers complementary to two adaptors. Following the purification of amplified cDNAs, the products were sequenced by using Hiseq2500 in Illumina Genome Analyzer (Illumina, San Diego, CA, USA). All sequencing reads were deposited in the Short Read Archive (SRA) database (http://www.ncbi.nlm.nih.goc/sra/), which are retrievable under the accession number (SRP054992).

### Bioinformatics analysis

After masking the adaptor sequences and removing the reads with excessively small tags or contaminated adapter-adapter ligation, the clean reads with 15–33 nt in length were processed for further bioinformatics analysis. Since *A*. *marmorata* lacks a reference genome, the remaining reads were mapped to European eel *Anguilla Anguilla* genome (http://www.zfgenomics.org/sub/eel), one of *A*. *marmorata* closely related species [34], with exact match in the seed region by using Bowtie software (parameters:–n, 0, -1 and 15) [35]. The reads mapped to the European eel *A*. *anguilla* genome were filtered to discard rRNA, tRNA, snRNA, ncRNA and other snoRNA sequences by BLAST against the NCBI Genbank database (www.ncbi.nlm.nih.gov/) and Rfam database (11.0, http://Rfam.sanger.ac.uk/).

The remaining sequences will be identified as conserved miRNAs in *A*. *marmorata* if these sequences exactly matched the conserved miRNAs with miRbase data (version 20.0, http://www.mirbase.org/) by using bowtie program (parameters:–n, 0, -1 and 15). In order to describe the nucleotide bias of identified miRNAs in *A*. *marmorata*, conserved miRNA indentified in our sRNA library will be used to count the nucleotide bias at each position.

The sequences will be identified as novel miRNAs in *A*. *marmorata* if they mismatched to conserved miRNAs with miRbase, but shared the same seed region with the conserved miRNA in miRbase by using miRDeep2 (mapper. pl config_miRDeep; parameters:-e,-d,-h,-i,-j,-l, 18,-m and-p). RNA-fold program was used to reveal the propensity of miRNA structures with the default parameters [36].

In order to explore the differential expression of mature miRNAs, the reading counts of conserved miRNAs in three libraries were used as the strategy to evaluate the relative abundance after normalization, which was conducted by using miRDeep2 quantifier. pl module (default parameters). In order to reveal the differential expression of pre-miRNAs in three libraries, the counts of the reads that matched with miRbase-annotated pre-miRNAs but not matched with mature miRNA in miRbase were used to calculate Fragments per Kilobase of transcript per million fragments mapped (FPKM). The FPKM expression was computed by using cufflink program with default parameters, and the FPKM score can response to the expression of known miRNA hairpins.

MiRanda program (parameters: S > 90 and ΔG < −17 kcal/mol) was utilized to clarify the functions of the identified miRNAs by predicting their target genes [37,38]. Furthermore, Gene ontology (GO) annotation and Kyoto Encyclopedia of Genes and Genomes (KEGG) pathway analysis were performed to identify the functional modules regulated by miRNAs.

### Quantitative real-time PCR

In order to validate and characterize the differentially expressed miRNAs in *A*. *marmorata* cultured in different salinities, the relative expression of 12 miRNAs including 8 known and 4 novel miRNAs was selected and analyzed by quantifying the miRNA stem-loop. Total RNAs were isolated using the same reagents as described above. Reverse transcription was performed in a 20-μL reaction system consisting of 1 μL of total RNA, 1 μL of enyzme mix, 1 μL of specific primer, 5 μL of 5× RT buffer and 12 μL of ddH_2_O using ReverTra Ace qPCR RT Kit (TOYOBO, Japan). Briefly, after a reverse transcription step at 42°C for 18 minutes and enzyme inactivation step at 85°C for 5 seconds, the cDNA was synthesized accordingly and the new synthesized cDNA was stored at -20°C for subsequent quantitative real-time PCR (qRT-PCR). QRT-PCR was performed on ABI Step One Plus system (Applied Biosystems, Foster, CA). The qRT-PCR experiments were performed in a 20-μL reaction system consisting of 2 μL of diluted cDNA template, 10 μL of 2× Realtime PCR Master Mix, 0.4 μL of each primer (10 mmol/μL) and 7.2 μL of ddH2O using SYBR Green Realtime PCR Master Mix (TOYOBO, Japan). The PCR amplification was conducted under an initial denaturation at 94°C for 30 seconds, and then 40 cycles of amplification including the denaturation at 94°C for 20 seconds, annealing at 61°C for 30 seconds, extension at 72°C for 30 seconds; after 40 cycles, final extension at 72°C for 1 minute. The specific RT primers and stem-loop primers are shown in supplementary data (S1 Table).

In order to explore the osmoregulatory roles of miRNAs, the temporal expression levels of 12 mature miRNAs were further examined. Total RNA was isolated. Subsequently, reverse transcription was performed in a 20-μL reaction system consisting of 1 μL of total RNA, 1 μL of miRNA RT enyzme mix, 10 μL of 2× TS miRNA Reaction Mix and 8 μL of ddH_2_O by using miRNA First-Strand cDNA synthesis Supermix (TransScript, Beijing, China). Briefly, after a reverse transcription step at 37°C for 1 hour and enzyme inactivation step at 85°C for 5 seconds, the cDNA was synthesized accordingly and the new synthesized cDNA was stored at -20°C for subsequent qRT-PCR. QRT- PCR was performed on ABI Step One Plus system (Applied Biosystems, Foster, CA). The qRT-PCR amplification was performed in a 20-μL reaction system consisting of 1 μL of diluted cDNA template, 10 μL of 2× Top Green qPCR superMix, 0.4 μL of each primer (10 mmol/μL), 0.4 μL of Passive Reference Dye (50×) (optional) and 7.8 μL of ddH2O by using Green miRNA qRT-PCR SuperMix (TransScript, Beijing, China). The PCR reactions were performed as follows: 94°C for 30 seconds, and then 40 cycles with 5 seconds at 94°C and 30 seconds at 60°C. The primers are shown in supplementary data (S2 Table).

Each qRT-PCR experiment was performed in triplicate, and each independent experiment was composed of three biological replicates. Finally, the default melting curve step in ABI Step One Plus system (Applied Biosystems, Foster, CA) was performed to verify the amplification specificity. U6 was used as an internal control. The expression of miRNAs was measured by using the 2^-△△CT^ method [39].

### Statistical Analysis

The data of qRT-PCR were expressed as Mean ± SD. Statistically significant difference was examined by a t-test through SPSS 13.0 software. The *p* value less than 0.05 was considered as the statistically significant difference.

## Results

### Features of sRNAs in *A*. *marmorata* cultured in different salinities

In order to identify miRNA differentiation of *A*. *marmorata* exposed to three different salinities, three sRNA libraries representing the gills of *A*. *marmorata* cultured in FW, BW and SW were constructed with total RNA and subjected to Illumina sRNA deep sequencing. In total, 8,928,604 and 6,924,130 raw reads were obtained from FWP1 and FWP2, 7,636,838 and 8,570,151 raw reads from BWP1 and BW0P2, 10,336,326 and 7,487,310 raw reads from SWP1 and SWP2, respectively.

After quality control, we obtained 6,289,961 and 5,049,207 clean reads with 15–33 nt from FWP1 and FWP2, 5,273,102 and 6,685,304 from BWP1 and BWP2, 7,574,411 and 4,994,553 from SWP1 and SWP2, respectively (S3 Table). Among these clean reads, 4,911,979 and 3,832,520 sequences from FWP1 and FWP2, 4,395,015 and 5,508,995 sequences from BWP1 and BWP2, 6,014,642 and 3,906,573 sequences from SWP1 and SWP2 matched perfectly to that of the European eel *A*. *anguilla* genome, with the similarity of 78.09%, 75.90%, 83.34%, 82.40%, 79.41% and 78.22% to the clean reads, respectively. In addition, *A*. *anguilla* genome also can be used to screen sRNAs from mRNA degradation pathways. These results showed excellent matching degree with exon sense, followed by matching intron sense in our six sRNA libraries. During the detection of repeat reads (download from RepBase http://www.girinst.org.), there were 1,785,829, 733,956 and 2,099,259 clean reads matched with repeat sequences in FW, BW and SW, respectively. The non-miRNAs were disclosed according to Rfam database, followed by a disposal of 362,703 and 196,749 reads from FWP1 and FWP2, 332,046 and 427,494 reads from BWP1 and BWP2, 574,914 and 435,468 reads from SWP1 and SWP2 (S4 Table).

**Fig 1:**
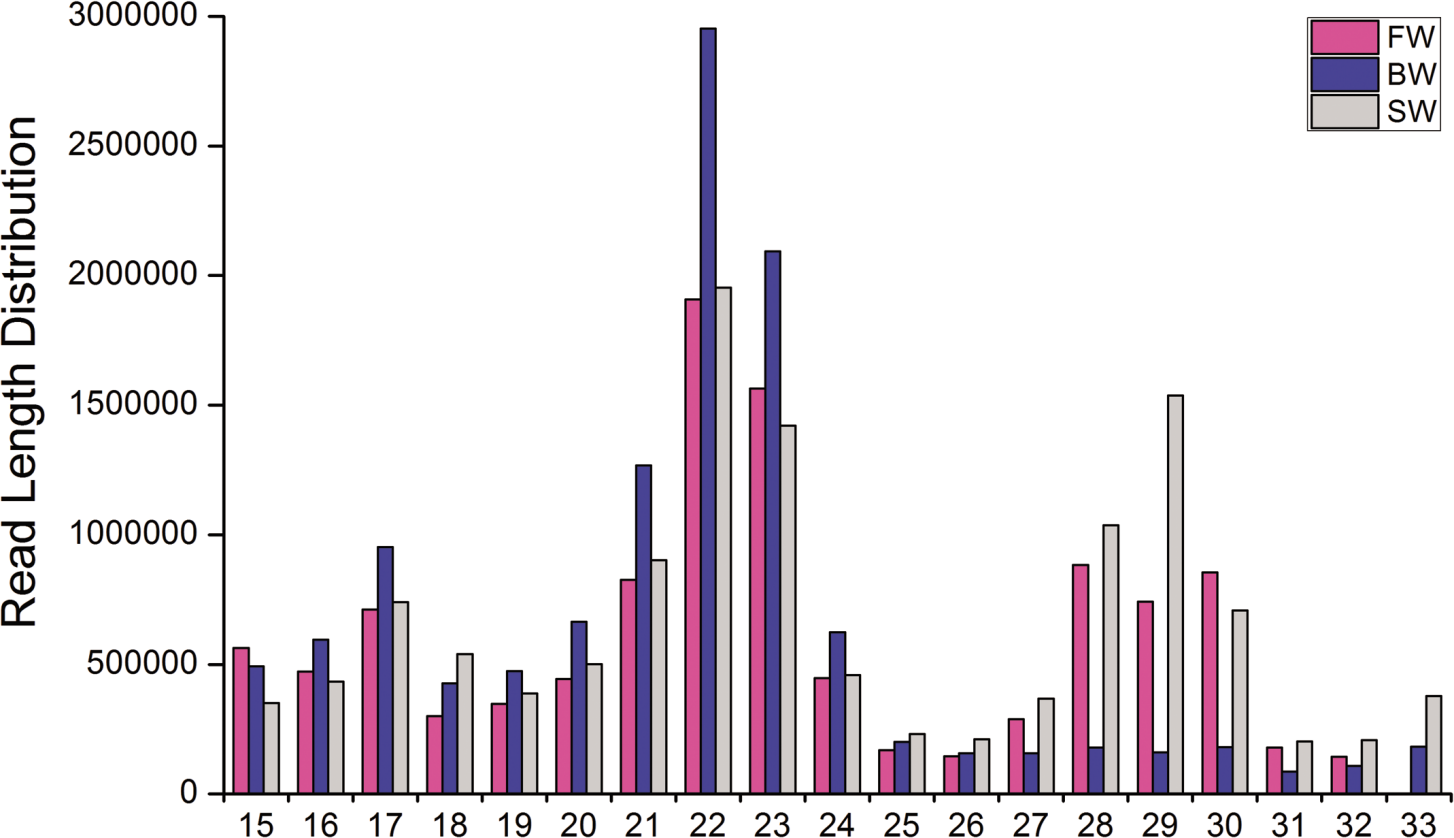
Length distribution of sRNA sequences of *A*. *marmorata* in three libraries. Sequence length distribution of clean reads based on the abundance; the most abundant size class was 22 nt in three libraries, followed by 23 nt in FW and BW but 29 nt in SW.

The sRNA-sequencing results indicated that 22 nt sRNAs were the most abundant, whose amounts were up to 16.82%, 24.70%, and 15.55% of the total sRNAs in FW, BW and SW, respectively. The second most abundant sRNA was 29 nt in SW, but was 23 nt in FW and BW, and with abundance of 28–30 nt sRNAs in FW and SW was higher than that in BW (Fig 1).

### Identification of conserved mature miRNAs in *A*. *marmorata*

The Illumina sRNAs deep sequencing approach allows us to determine the relative abundance of various miRNA by calculating the sequencing frequency. As a result, 34 conserved miRNAs were found in our sRNA libraries. A highly expressed miRNA may have a large number of sequenced clones. The miRNAs were considered as eligible for differential expression analysis when normalized expression (NE) is larger than 1 in all salinities, otherwise clean reads were ignored. A number of mature miRNAs such as miR-10b-5p, 181a, 26a-5p, 30d, and 99a-5p exhibited a broad range of expression levels by abundantly expressing more than hundreds of thousands of sequence reads in all salinities. Among them, miR-10b-5p is the most abundant miRNA; on the contrary, some miRNAs such as miR-1a-2-5p, miR-727-5p and miR-466k showed less than 10 reads (S5 Table). The different categories and the expression of miRNAs often reflect the different roles in a particular tissue or development stage as well as corresponding to biological mechanisms.

### Nucleotide bias of conserved mature miRNAs in *A*. *marmorata*

**Fig 2:**
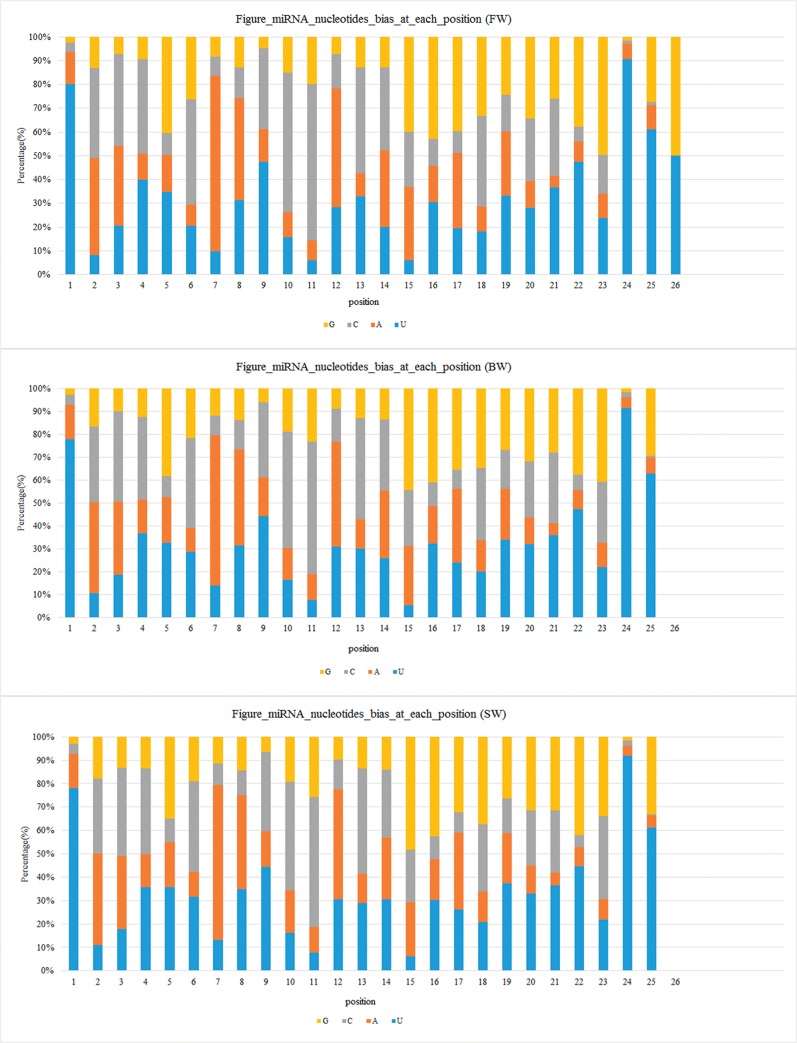
Nucleotide bias of conserved miRNAs at each position of *A*. *marmorata* in three libraries. The most frequent nucleotide in the first nucleotide and the ninth nucleotide at the 5’ end is U. (A) Nucleotide bias of conserved miRNAs at each position in FW. (B) Nucleotide bias of conserved miRNAs at each position in BW. (C) Nucleotide bias of conserved miRNAs at each position in SW.

Basic compositions of miRNAs are one of the most fundamental features of miRNA sequences, especially the first nucleotide bias in miRNAs. In the present study, we analyzed the 1st nucleotide bias and each position of mature miRNAs, which matched perfectly to miRbase known miRNAs in our three libraries. As a result, uridine (U) was the most frequent nucleotide (mean = 64.65%) as the first nucleotide at the 5’ end in conserved miRNAs of *A*. *marmorata* (Fig 2 and S6 Table). The phenomenon of nucleotide bias may be correlated with the mechanisms of miRNA actions, such as binding with the targets for gene regulation. Also, the ninth nucleotide in the 5’ end is highly enriched by U. Therefore, the 5’ and 3’ edges of the seed region [40,41], known to have a critical role in targeting miRNA to mRNA for translational inhibition or mRNA cleavage, are flanked by U. The nucleotide bias analysis at each position has revealed that U and guanine (G) are mainly located at the beginnings and the ends of the reads (Fig 2).

### Identification of conserved pre-miRNAs in *A*. *marmorata*

The Illumina sRNA-seqencing approach also allows us to determine the relative abundance of various pre-miRNAs by calculating the FPKM score. Those pre-miRNAs that have been fully sequenced for read coverage can be used for relative abundance analysis. As a result, 184 known pre-miRNAs were used for the assessment of miRNA expression analysis (status as OK in miRdeep2 quantifier.pl with the default parameters). The most abundant pre-miRNA was mir-205a with FPKM scores of more than one hundred million in all salinities, while miR-92a, miR-10b, miR-181, miR-92b, miR-26a, miR-99a and miR-454 showed predominant expression with more than 200,000 FPKM scores (S7 Table).

### Identification of novel miRNAs in *A*. *marmorata*

During searching of novel miRNAs, the mapped reads excluding known miRNAs were evaluated by miRDeep2 and RNA-fold. As a result, 613 novel miRNAs were predicted with total read counts varying from 263371 to 3; additionally, their miRDeep2 scores were diverged from 854020.6 to 0, and the estimated probability that the miRNA candidate is a true positive is ranged from 97 ± 1% to 57 ± 3%. RNA-fold was implemented to predict potential precursor of miRNA structure and the *p* values of 523 of 613 predicted miRNA structures were reported as the significant (*p* < 0.05). Notably, 519 of 613 predicted novel miRNAs carried with the same seed with known miRNAs in miRbase database (S8 Table), indicating that these miRNAs may be the new members to the known miRNA families.

### Differential expression of conserved mature miRNAs in eels cultured in different salinities

**Fig 3:**
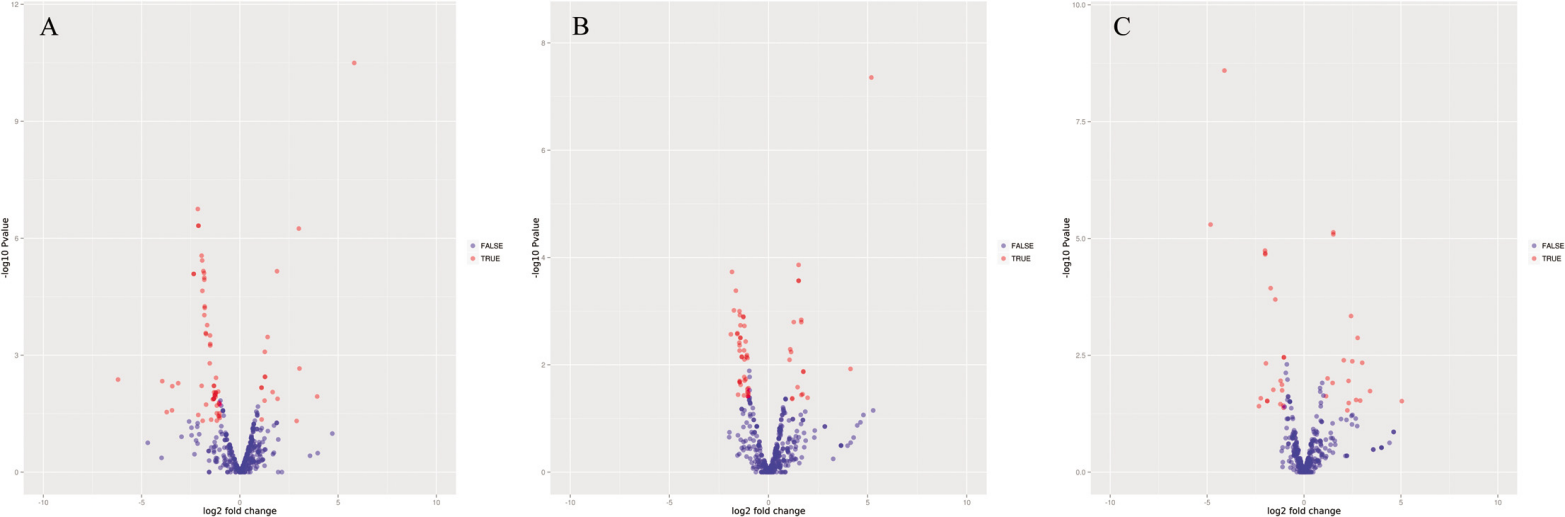
Difference of mature miRNA expression in BW compared with FW, in SW compared with FW and in SW compared with BW. Volcano plot of miRNA expression levels in BW compared with FW (A), in SW compared with FW (B) and in SW compared with BW (C). Each point represents a miRNA. Blue points represent significantly differentially expressed miRNAs.

**Fig 4:**
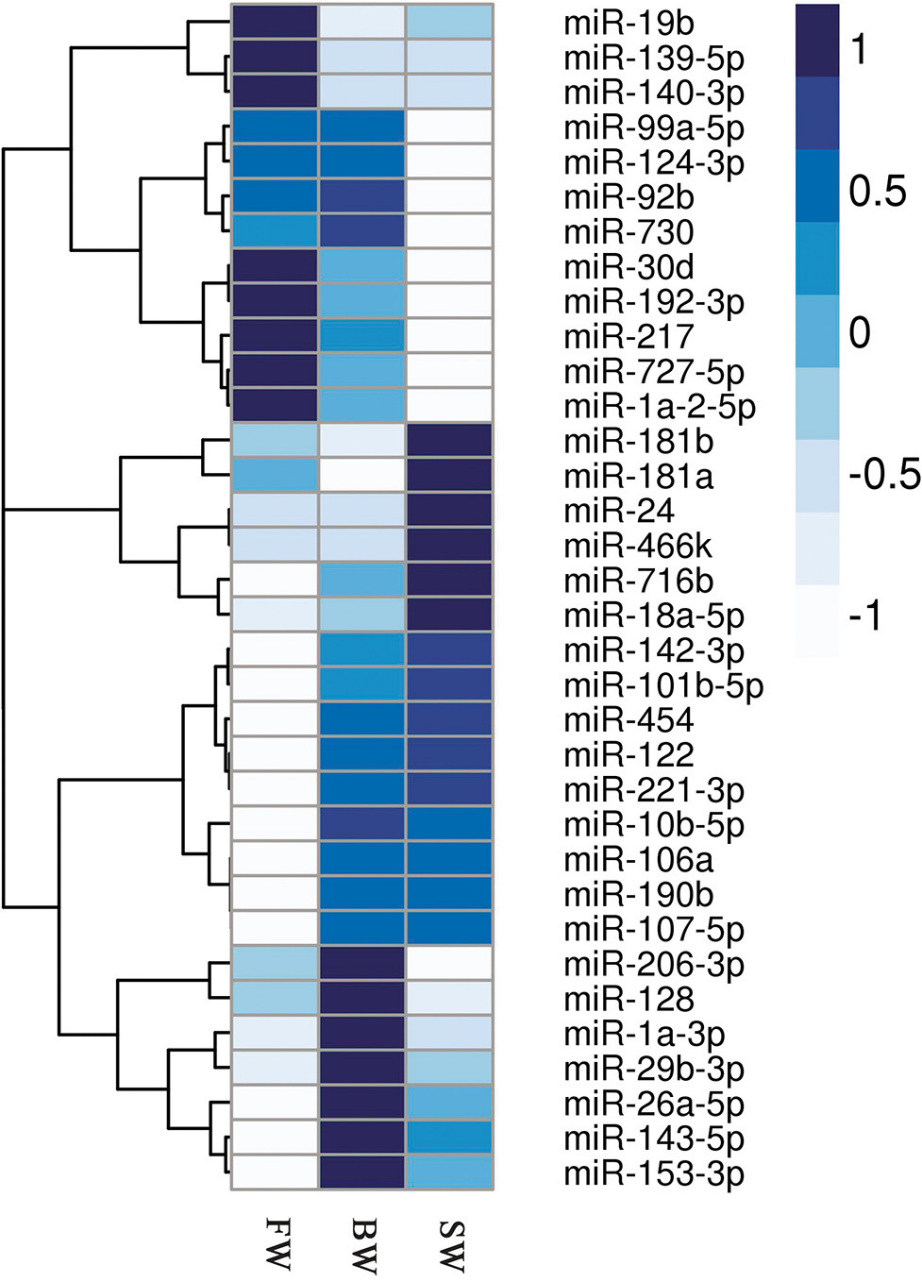
Hierarchical clustering of conserved miRNAs differentially expressed in three different salinities. The heat map is drawn with log_2_(NE+1) of each miRNA. Color map is used to distinguish the difference in the expression of miRNAs.

The major objective of the present study is to illustrate the differential expression in *A*. *marmorata* cultured in different salinities. Based on the deep sequencing results, the relative expression levels of miRNAs could be calculated. Totally 29 miRNAs were significantly up-regulated, while 72 miRNAs were significantly down-regulated in eels exposed to BW compared with the eels exposed to FW. Similarly, 24 miRNAs were significantly up-regulated, while 54 miRNAs were significantly down-regulated in eels exposed to SW compared with the eels exposed to FW. In addition, 24 miRNAs were significantly up-regulated, while 45 miRNAs were significantly down-regulated in eels exposed to SW when compared with the eels exposed to BW (*p <* 0.05) (Fig 3). The up-regulated miRNAs such as miR-122 and miR-190b showed 5-fold and 4-fold higher expression in SW than that in FW. In contrast, miR-124-3p, the most down-regulated miRNAs, showed 10-fold higher expression in SW than that in BW, while miR-1a-3p and miR-206-3p exhibited 2-fold increase. Interestingly, there was no significantly up-regulation for known mature miRNAs in SW to BW (Fig 4 and S9 Table).

**Fig 5:**
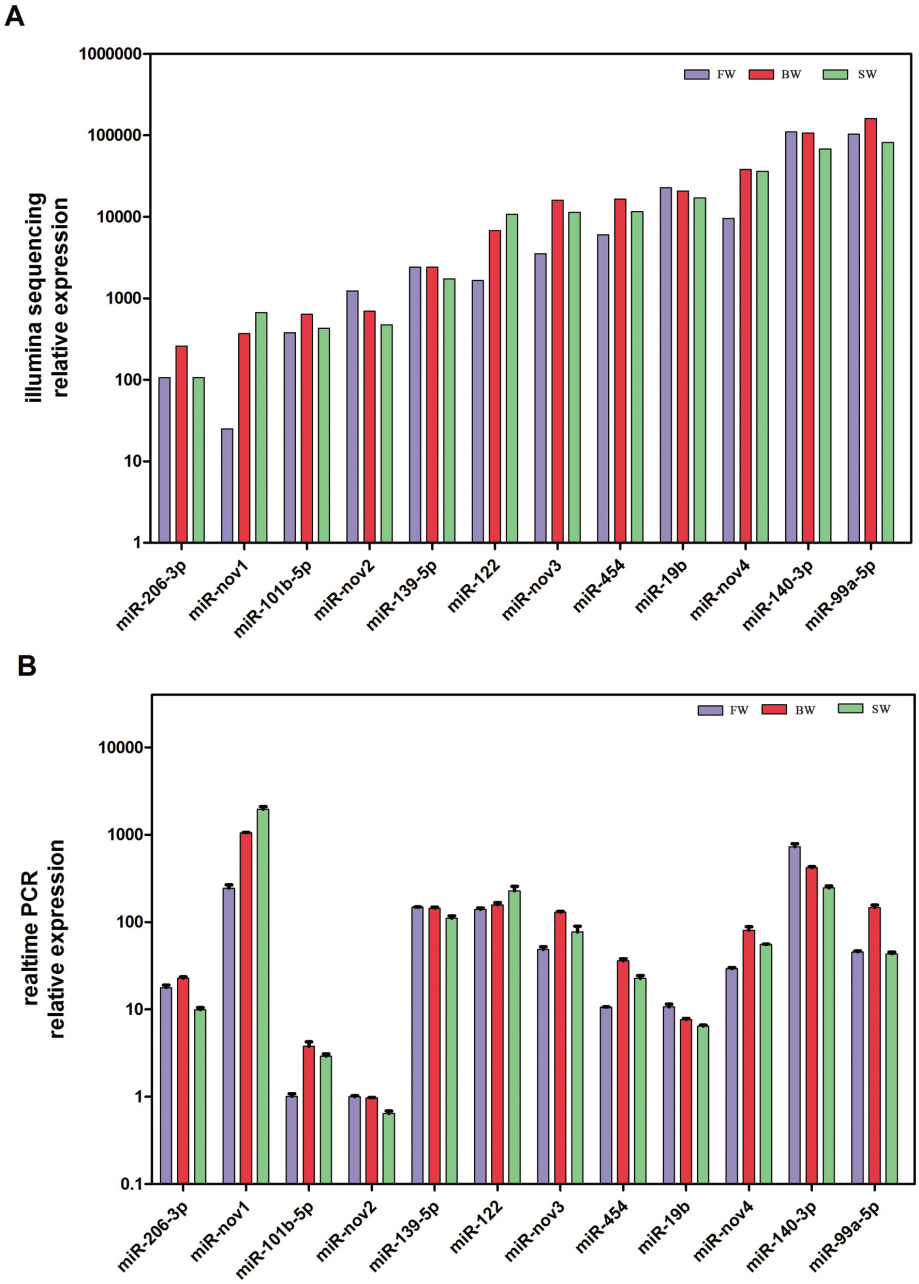
Quantitative real-time PCR validation of differentially expressed miRNAs identified using Illumina sRNA deep sequencing. (A) Profile of sequencing frequencies for miRNAs in different salinities; (B) Profile of relative expression of miRNAs evaluated by qRT-PCR.

In order to validate the differential expression, 12 mature miRNAs composed of 8 significantly differentially expressed mature miRNAs (including 4 known miRNAs: miR-139-5p, miR-140-3p, miR-19b and miR-122, and 4 novel miRNAs: miR-nov1, nov2, nov3 and nov4) and 4 similarly expressed mature miRNAs including miR-99a-5p, miR-454, miR-101b-5p and miR-206-3p were assayed by qRT-PCR (Fig 5). The relative expression of 11 miRNAs was consistent with the Illumina sequencing results, except for a slight difference with miR-206-3p due to the mismatching by primer-miRNA binding.

### Differential expression of conserved pre-miRNAs in eels exposed to different salinities

**Fig 6:**
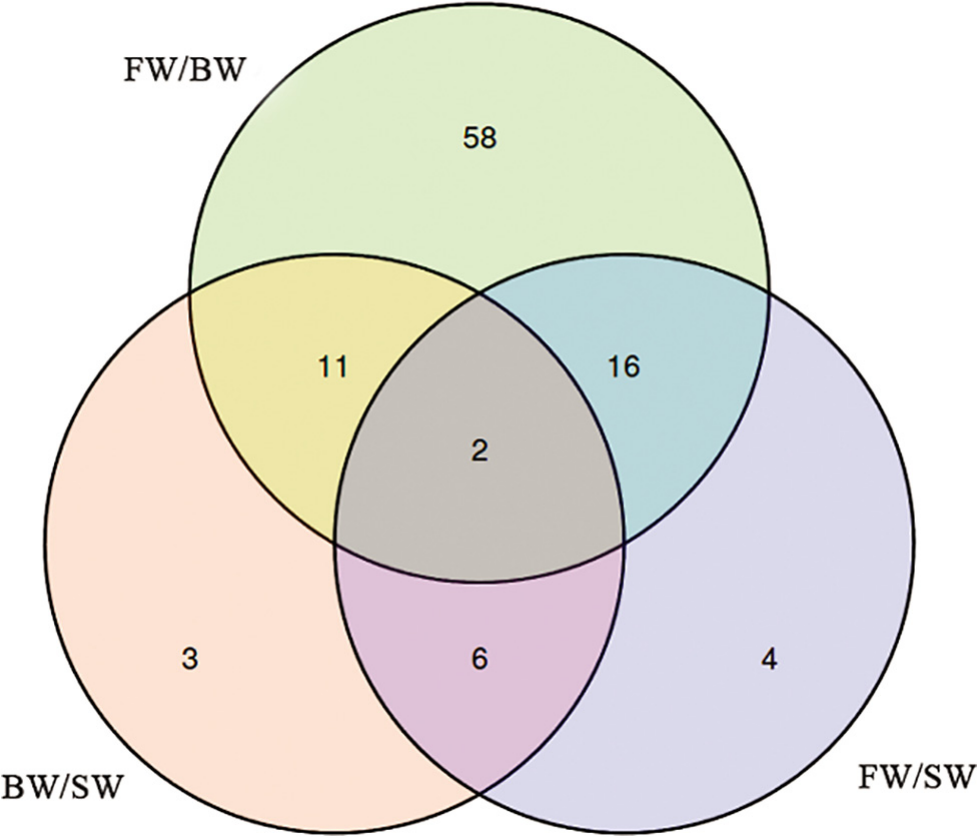
Venn diagram comparing the expression distribution of miRNAs in BW compared with FW, in SW compared with FW and in SW compared with BW. Numbers in parentheses represent the numbers of co-expressed or differentially expressed pre-miRNAs.

The relative expression of known miRNA hairpins was calculated on the basis of their FPKM scores. Totally 184 conserved pre-miRNAs were found in all salinities, 166 of 184 pre-miRNAs were co-expressed. As a result, 26 known pre-miRNAs such as miR-122, miR-429, miR-454b, miR-30e and miR-33a significantly up-regulated (*p <* 0.001) in eels exposed to BW compared with those of FW. Similarly, miR-122 and 190b were significantly up-regulated and miR-103 was significantly down-regulated in eels exposed to SW compared with those of FW (*p <* 0.05). MiR-21-1 was significantly up-regulated, while miR-203 was significantly down-regulated in eels exposed to SW compared with the eels exposed to BW (*p <* 0.001) (S7 Table). Particularly, the significantly differential expression of 58, 4 and 3 conserved pre-miRNAs were observed in FW compared with BW, in FW compared with SW and in BW compared with SW, respectively, while only 2 pre-miRNAs were significantly differential expression in all salinities (Fig 6).

### Osmoregulatory expression patterns of miRNAs in eels exposed to different salinities

All results above showed that the approach using sRNA sequencing is a reliable and effective method for identifying miRNA expression in *A*. *marmorata* cultured in different salinities. In order to investigate whether miRNAs play the osmoadaptation role in different salinities, the temporal expression levels of 12 mature miRNAs were further examined using qRT-PCR in FW (0 h) as the control, and 1, 6, 12 and 24 h after exposed to BW and SW. These 12 miRNAs including miR-10b-5p, miR-181a, miR-181b, miR-26a-5p, miR-99a-5p and miR-454 were dominantly expressed, and miR-139-5p, miR-140-3p, miR-19b, miR-122, miR-30d and miR- 92b were significantly differentially expressed.

**Fig 7:**
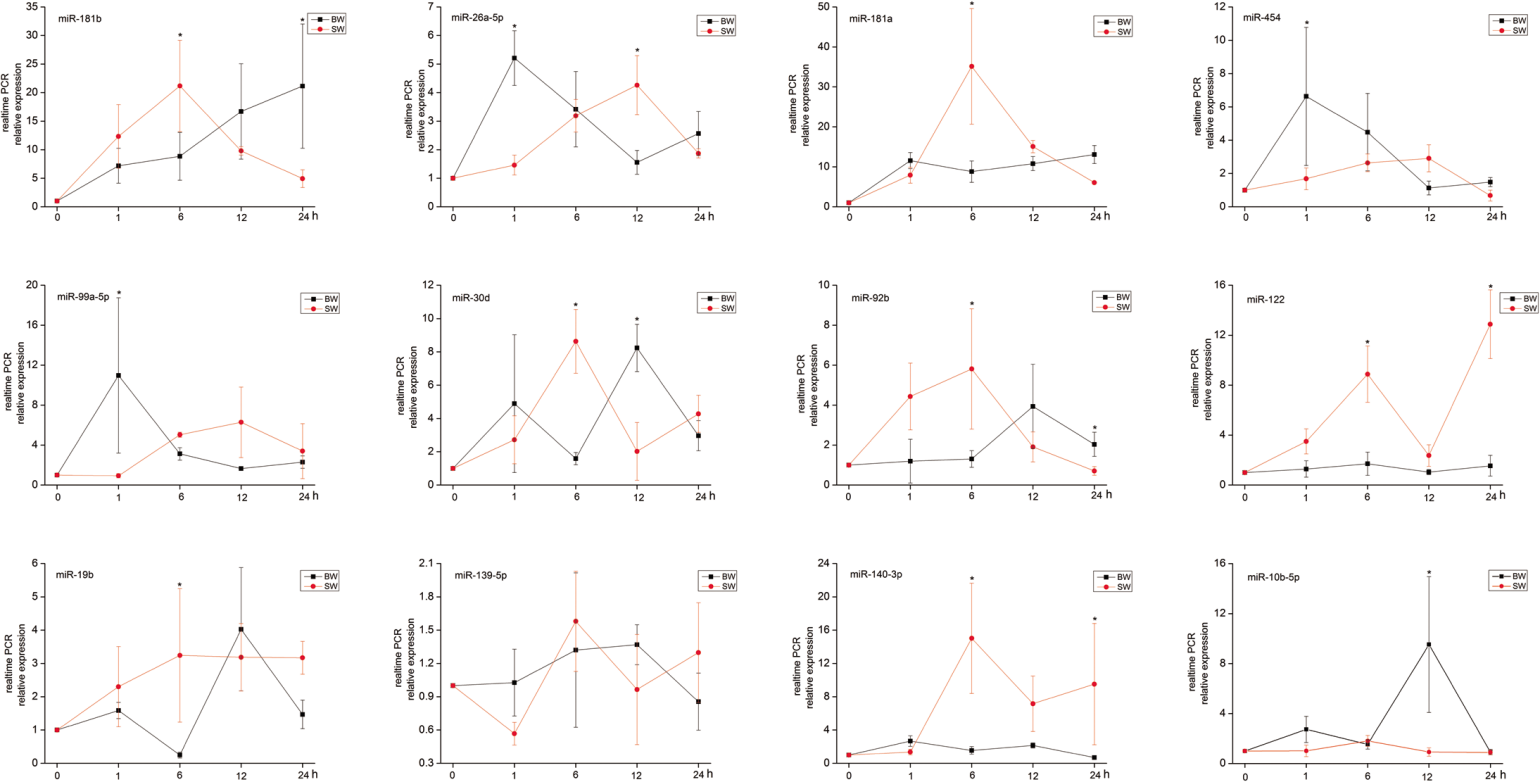
Expression patterns of miRNAs in the gills at different time points. Expression of miR-181b, miR-26a-5p, miR-181a, miR-454, miR-99a-5p, miR-30d, miR-92b, miR-122, miR-19b, miR-139-5p, miR-140-3p and miR- 10b-5p were assayed by qRT-PCR. *Significant difference between BW and SW (*p* < 0.05).

The results demonstrated differential expression patterns of miRNAs in different time points when transferred to BW and SW. For instance, the expression of miR-122 and miR-140-3p was similar, and these 2 miRNAs almost did not reveal any change in their expression within 24 h after transferred to BW from FW, but the expression was increased in 1 h and 6 h then decreased in 12 h and increased again in 24 h when transferred to SW from FW. On the contrary, the expression of miR-10b-5p did not reveal any change within 24 h when transferred to SW from FW, but reached its peak level in 24 h when transferred to SW from FW. The other 9 miRNAs were differentially expressed in BW compare with FW and in SW compared with FW (Fig 7). The expression patterns of these 12 miRNAs suggest that the miRNAs may regulate the response to osmotic stress variably.

### Target prediction and function annotation

**Fig 8:**
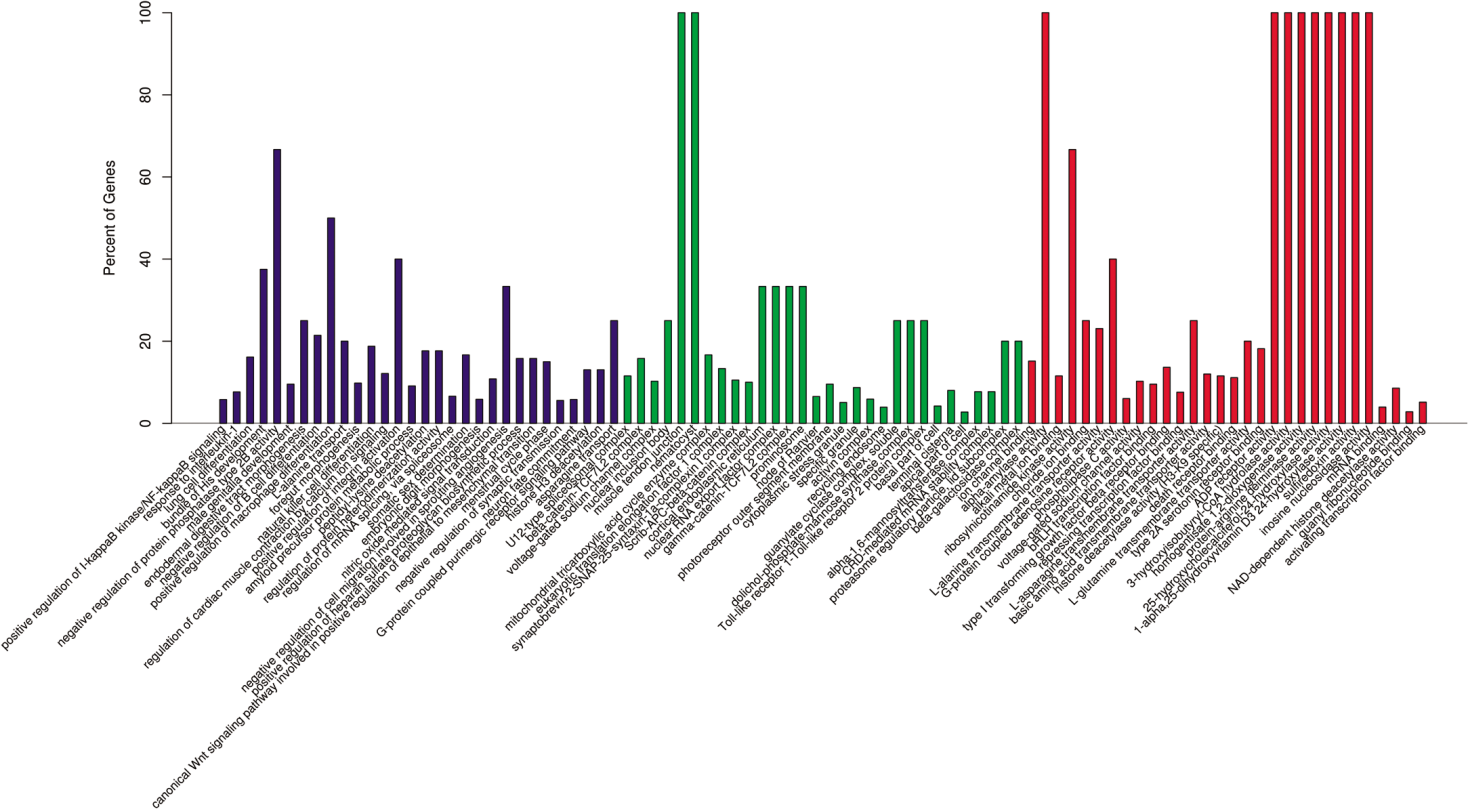
Gene ontology (GO) classification annotated for predicted target genes of differentially expressed miRNAs. Partial GO enrichment for the predicted target genes is shown in biological processes (blue part), cellular compartments (green part) and molecular functions (red part).

**Fig 9:**
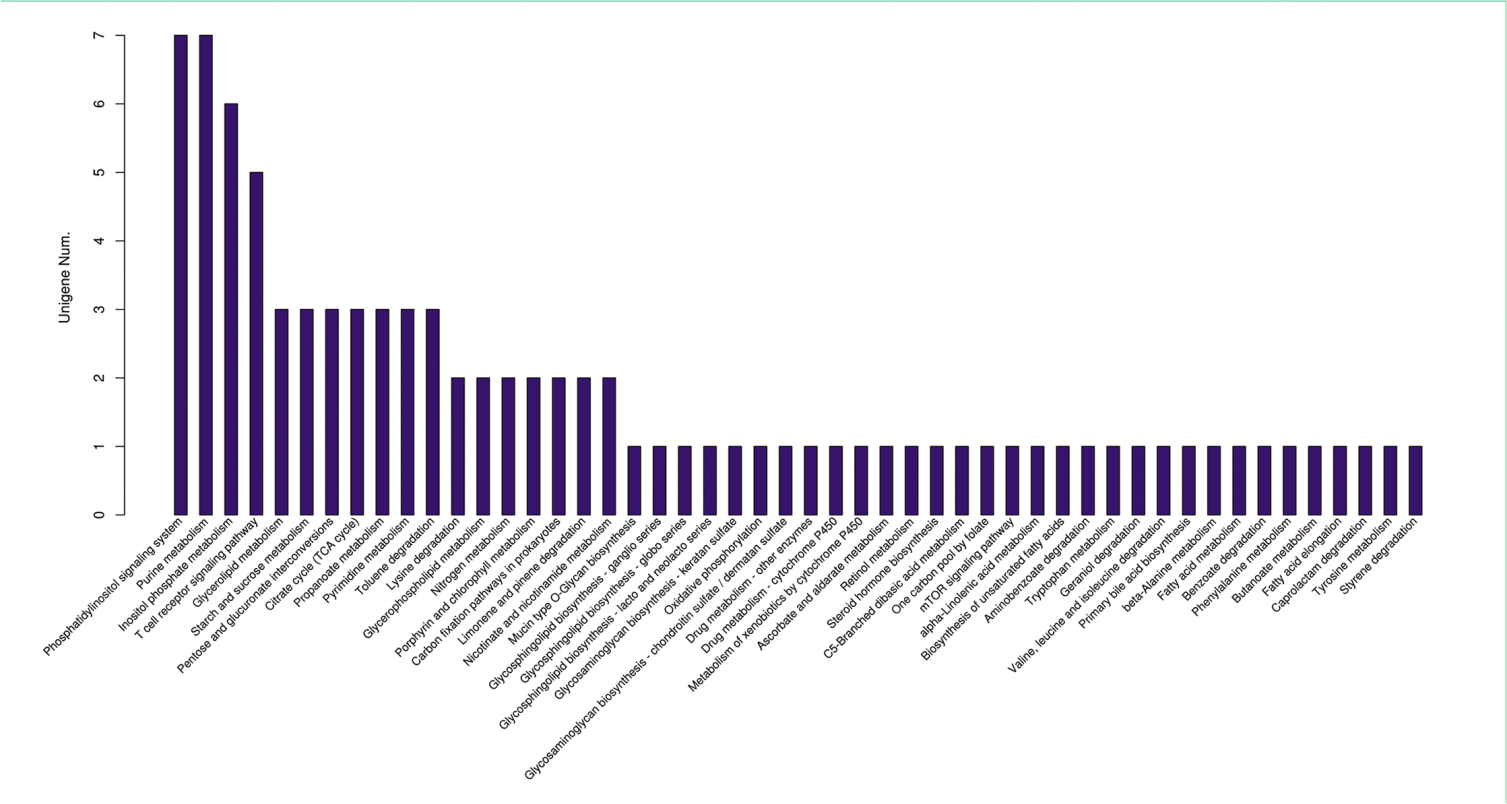
Summary of KEGG pathway enrichment for predicted target genes of differentially expressed miRNAs.

The determination of physioregulatory properties of miRNA is elucidated by the prediction of target genes of significantly differentially expressed miRNAs (*p* < 0.05) between salinity sets using miRanda. In total, 773 target genes were found (data not shown). The predicted target genes were further categorized through GO annotation and KEGG pathway analysis. After analyzing the top 30 most enrichment GO annotation, the most abundant gene counts were shown in negative regulation of protein phosphatase-type 2B activity (GO:0032513) GO term in biological process and 2 GO term including muscle tendon junction (GO:0005927) and nematocyst (GO:0042151) in cellular compartment level. Notably, in molecular function level, there are 8 GO terms including 3-hydroxyisobutyryl-CoA hydrolase activity (GO:0003860), homogetisate 1, 2-dioxygenase activity (GO:0004411), protein-arginine deiminase activity (GO:0004668), 25-hydroxychlecalciferol-24-hydroxylase activity (GO:0008403), 1-alpha-25-dihydroxyvitamin D3 24 hydroxylase activity (GO:0030342), sulfiredoxin activity (GO:0032542) and inosine nucleosidase activity (GO:0047724) associated with most abundant gene counts (Fig 8). Subsequently, the KEGG pathway analysis revealed two major pathways occupied by the most abundant gene counts of significantly differentially expressed miRNAs including phosphatidylinositol signaling system (Ko04070) and purine metabolism (Ko00230) (Fig 9). The crucial deviation in the number of the target gene counts implied that the varied levels of miRNAs involved in these GO terms and pathways.

## Discussion

The marbled eel, *A*. *marmorata*, is one of the important economic fish in Southeast Asia, widely spread across tropical and subtropical oceans and associated with fresh water systems. In the present study, a comprehensive annotation and analysis of the miRNAs expressed in *A*. *marmorata* exposed to different salinities has been constructed. The analysis for the length distribution of sequenced sRNAs has been illustrated that the dominant size of sRNAs in all salinities is 22 nt, followed by 23 nt and 21 nt. In addition, the length distribution of 28–30 nt sRNAs in FW and SW is higher than that in BW. This feature is consistent with the fish species such as blunt snout bream, tilapia, bighead carp, silver carp, *Pseudosciaena crocea*, *Paralichthys olivaceus* and *Cynoglossus semilaevis* [42–47], but not with other vertebrates such as dairy goat and swine [48,49]. This phenomenon suggests that the length distribution may be similar in closely related fish species. Up to now, the data of sRNAs in the gills of fish including *A*. *marmorata* are still limited, especially the information about sRNAs in different salinities is still rare. The length distribution in the gills of *A*. *marmorata* cultured in different salinities is urgently needed to be unveiled in the future.

The base compositions of miRNAs can influence their physiochemical and biological properties through affecting base pairing and the thermodynamic folding of miRNA secondary structure [48], therefore, a configuration change in the structures of miRNAs can adversely alter their activities [50–52]. The U, as the most common base at the 5’ end in miRNAs, is substantiated by several studies [6,53]. In our sRNA libraries, the most frequently nucleotide in the first nucleotide and the ninth nucleotide at the 5’ end is U. This feature suggests that U is selectively favored at the seed region, which may account for its prominent functions in miRNA biogenesis and mRNA target recognition.

In all salinities, the most abundant sequenced mature miRNAs are miR-10b-5p, miR-181a, miR-181b and miR-26a-5p that are expressed more than hundreds and thousands of sequence reads. MiR-181 family is known for its ability to alter cellular metabolism and to regulate survival, organism size, and PTEN expression in thymocytes [54]. Similarly, miR-26a has been identified in the glomeruli as the contributor of renal failure [55], which is also required for the differentiation and regeneration of skeletal muscle [56]. However, there is no direct evidence for supporting the involvement in osmoregulation of these miRNAs.

The differentially expressed miRNAs such as miR-122, 190b, 124-3p, 1a-3p and 206-3p showed a potential role in osmoregulation when they are either significantly up-regulated or down-regulated in different salinities. As the liver-specific miRNA, miR-122 can regulate lipid metabolism [57,58], which is an major regulator of cellular energy metabolism [10]. In hepatocellular carcinoma, miR-190b is effective in the suppression of IGF-1 [59], and it is reported to play a critical role in fish osmoregulation [24]. Interestingly, miR-190b is one of the molecular targets of polyphenols [60], and exhibits a variety of anti-carcinogenic effects on the prevention of angiogenesis [61]. *In vitro* luciferase assays, miR-124 can bind to the target sequence located in the 3’-UTR of the mineralocorticoid receptor (Nr3c2) [62]. These studies in tilapia have unraveled that its growth is regulated by miR-206 through modulating IGF-1 gene expression; in contrast, the loss of miR-206 function leads to the accelerated growth [30].

The GO annotation and KEGG pathway analysis was carried out to identify the predicted target gene of significantly differential expressed miRNAs. Negative regulation of protein phosphatase-type 2B activity acted as the most abundant gene count GO term in biological process. In the previous studies, phosphatase is considered an important indicator of calcium metabolism and osmoregulation in Atlantic salmon [63]; protein phosphatase also can inhibit Na^+^/H^+^ exchanger in *Pleuronectes americanus* and then affect its osmoregulation [64], indicating the significance of phosphatase regulation in fish osmoregulation. Some evidences have demonstrated that hydroxylase-related genes specific to steroidogenic interregnal tissue are also expressed in renal tissues [65,66]. The hydroxylation of vitamin D plays an important role to maintain fish plasma levels and protein-bound transport in blood plasma [67]. In another hand, 25-hydroxychlecalciferol-24-hydroxylase activity and 1-alpha-25-dihydroxyvitamin D3 24 hydroxylase activity act as the most abundant target gene count GO term in molecular function level. Phosphorylation of the transporter acting inhibitory and dephosphorylation leading to activation/inactivation in fish cells, and phosphatidylinositol-mediated exocytic insertion of the transporter into the membrane can execute a vital role in fish physiology [68]. All these results above revealed the potential osmotic regulatory function of the differential expressed miRNAs in the three libraries.

In order to investigate whether miRNAs play the osmoregulatory roles in different salinities, temporal expression patterns of 12 miRNAs have been evaluated by qRT-PCR. We have selected 12 miRNAs for further examination in 9 different time points. Interestingly, miR-122 and 140-3p demonstrated osmoregulatory effects in SW, while miR-10b-5p showed osmoregulatory effects in BW. This phenomenon suggests that these three miRNAs may have different roles in osmotic regulation. Other 9 miRNAs exhibited differential expression, suggesting that these 9 miRNAs may have potential effects on osmoregulation. Even though there are some studies regarding to differential expression of miRNAs in response to different osmotic pressure [27,28], the expression of miRNAs in different salinities are rarely reported, especially in fish.

In the present study, we have demonstrated the differential expression patterns of miRNAs subjected to various salinities, and pinpointed a variety of miRNAs with respect to fish osmoregulation. For future perspective, the subsequent studies for elucidating the possible osmoregulatory mechanisms of miRNAs have been highlighted in this study.

## Supporting Information

S1 Table The primers used in the study using TOYOBO. (XLS)

S2 Table The primers used in the study using TransScript. (XLS)

S3 Table Overview of reads from raw data to high quality reads (clean reads). (XLS)

S4 Table Summary of Illumina sRNA deep sequencing data for clean reads in 3 salinities. (XLS)

S5 Table The expression patterns of conserved mature miRNAs in three salinities. (XLS)

S6 Table The first nucleotide bias of mature miRNAs in *A*. *marmorata*. (XLS)

S7 Table The expression patterns of known pre-miRNAs in different salinities. (XLS)

S8 Table The novel miRNAs in different salinities. (XLSX)

S9 Table The different expression of conserved miRNAs in different salinities. (XLS)
